# The NOTCH-HES-1 axis is involved in promoting Th22 cell differentiation

**DOI:** 10.1186/s11658-021-00249-w

**Published:** 2021-02-23

**Authors:** Chong Zeng, Zhongbao Shao, Zibo Wei, Jie Yao, Weidong Wang, Liang Yin, Huixian YangOu, Dan Xiong

**Affiliations:** 1 Medical Research Center, Shunde Hospital, Southern Medical University (The First People’s Hospital of Shunde), Foshan, 528300 China; 2 Department of Electronic Information Engineering, Guangzhou College of Technology and Business, Foshan, China; 3 Department of Hepatobiliary Surgery, Shunde Hospital, Southern Medical University (The First People’s Hospital of Shunde), Foshan, 528300 China; 4 Department of Endocrinology, Shunde Hospital, Southern Medical University (The First People’s Hospital of Shunde), Foshan, 528300 China; 5 Department of Anesthesiology Operating Room, Shunde Hospital, Southern Medical University (The First People’s Hospital of Shunde), Foshan, 528300 China; 6 Department of Hematology, Shunde Hospital, Southern Medical University (The First People’s Hospital of Shunde), Foshan, Guangdong China

**Keywords:** CD4^+^ T cells, NOTCH signaling, HES-1, Th22, Differentiation

## Abstract

**Background:**

NOTCH signaling has been shown to play a role in the production of interleukin-22 (IL-22) by CD4^+^ T cells. Multiple T-helper (Th) cell populations secrete IL-22. Th22 (CD4^+^IL22^+^IFNγ^−^IL17A^−^) cells are a subgroup of CD4^+^ effector T cells that primarily generate IL-22. The regulatory mechanisms of the NOTCH signaling pathway involved in differentiation of the Th22 cell subset have not been completely elucidated. This study aimed to further explore the involvement of NOTCH signaling in Th22 differentiation.

**Methods:**

*In vitro* combination of IL-6, IL-23, and tumor necrosis factor-α (TNF-α) treatment with naïve CD4^+^ T cells established the Th22 cell induced model. NOTCH signaling was activated by jagged-1 and inhibited by (2S)-N-[(3,5-difluorophenyl) acetyl]-L-alanyl-2-phenyl]glycine 1,1-dimethylethyl ester (DAPT). HES-1 siRNA and HES-1 vector were employed to knock down and induce overexpression of HES-1 to investigate the effect of NOTCH signaling on the differentiation of CD4^+^T cells into Th22 cells.

**Results:**

We observed that the proportion of Th22 cells, along with *Hes-1*, *Ahr*, and *Il-22* mRNA and protein expression, was increased by both jagged-1 and overexpression of HES-1. On the other hand, after the combined cytokine treatment of cells, and exposure to jagged-1 and DAPT or HES-1 siRNA, there was a decrease in the Th22 cell proportion, mRNA and protein expression of HES-1, AHR, and IL-22.

**Conclusions:**

Our study demonstrates that HES-1 enhancement in AHR and IL-22 up-regulation of NOTCH signaling can promote the skewing of naïve CD4^+^T cells toward Th22 cells. Also, the results of our study show that HES-1 is a crucial factor in Th22 cell differentiation.

## Background

Naïve CD4^+^T cells polarized into various effector T-helper (Th) cell subsets are characterized by the production of different cytokines and functions through signature transcription factors and cytokines [1]. Previous studies have shown that naïve CD4^+^ T cells can differentiate into Th1, Th2, Th9, Th17, Th22, regulatory T (Treg), and follicular helper T (Tfh) cells based on their respective transcription factors, environmental cues including cytokines, and ligand-receptor interactions from cell-cell contact [2–5]. For instance, naïve CD4^+^T cells can differentiate and develop into Th17 cells when treated with IL-6, TGF-β, IL-1β, and IL-23 via the transcription factor retinoid-related orphan receptor-γt (RORγt) [6], and release IL-17A, IL-17F, IL-21, and IL-22, which are involved in various autoimmune diseases. Additionally, naïve CD4^+^ T cells were stimulated by TGF-β via Foxp3 skewing in Treg production of IL-10, which inhibited inflammation [7]. After naïve CD4^+^ T cells are exposed to IL-6 and TNF-α, the Th22 cells are induced, and the transcription factor aryl hydrocarbon receptor (AHR) is activated [8–11]. Th22 secretes a primary cytokine, IL-22, which is crucial for maintaining skin immunity [12], mucosal antimicrobial host defense, and autoimmune disease [11, 13]. In addition to these classical key cytokines that induce naïve CD4^+^ T cells into different Th subsets, other factors such as NOTCH signaling also contribute to Th differentiation [14].

Cell-cell communication plays an essential role in metazoan development and is mediated by the NOTCH signaling pathway [15]. In mammalian cells, there are four highly conserved trans-membrane receptors, i.e. NOTCH-1, -2, -3, and − 4, and five ligands, i.e. jagged-1, -2, delta-like-1, -3, and − 4 [16]. The NOTCH signaling is initiated from two adjacent cells, and involves NOTCH ligands and receptors that are noncovalently bound. In the transmembrane region of NOTCH proteins, two successive cleavages occur at S2 and S3 sites by disintegrin and metalloproteinase (ADAM) and γ-secretase, respectively. This process triggers the release of the NOTCH intracellular domain (NICD) into the cytoplasm. Then, the NICD travels to the nucleus and activates the target genes hairy and enhancer of split 1 (HES-1), HES-1 related with the YRPW motif (Hey-1), cyclin D1 and p21. These genes mediate multiple cellular processes such as cell differentiation, migration, proliferation, apoptosis, and the cell cycle [17]. The NOTCH signaling pathway is blocked by γ-secretase, in turn inhibiting downstream target gene expression, and suppressing cell growth and proliferation [18, 19]. On the other hand, activation of NOTCH signaling promotes proliferation of breast cancer cells and differentiation of stem cells [20, 21]. Also, NOTCH signaling has been shown to play an essential role in maintaining cell fate decisions.

NOTCH signaling is important in the development and differentiation of T cells [21, 22]. This signaling is the interplay with signal transducer and activator of transcription 3 (STAT3) and mediates cell differentiation [23–25]. A previous study suggested that the NOTCH-AHR-IL-22 axis is involved in the pathogenesis of lung adenocarcinoma [26]. Similarly, NOTCH signaling has also been shown to promote secretion of interleukin (IL)-22 in CD4^+^ T cells [14, 27]. However, IL-22 is released from both Th17 and Th22 cells during cytokine production [9]. Further, how the NOTCH signaling regulates this process, and the molecular mechanisms involved in Th22 cell differentiation, are not clearly understood. In this study, we aim to better understand the mechanism of the NOTCH signaling pathway in the differentiation of naïve CD4^+^T cells into Th22 cells.

## Materials and methods

### Reagents

Roswell Park Memorial Institute (RPMI) 1640 medium and fetal bovine serum (FBS) were purchased from Gibco (Gaithersburg, MD, USA). Jagged-1 and ((2S)-N-[(3,5-difluorophenyl)acetyl]-L-alanyl-2-phenyl]glycine-1,1-dimethylethyl ester) DAPT were purchased from R&D (Minneapolis, MN, USA). Anti-CD4-conjugated fluorescein isothiocyanate (FITC), anti-CD62L-conjugated eFluor 450, anti-IL-17A-conjugated PE, anti-IL-22-conjugated APC, anti-IFN-γ-conjugated PerCP-Cyanine5.5, anti-CD3 and anti-CD28 monoclonal antibodies, brefeldin A, fixation/permeabilization buffer, and Trizol reagent were obtained from Invitrogen (CA, USA). IL-6, TNF-α, IL-23, anti-IL-4 antibody, and anti-IFN-γ antibody were purchased from Peprotech (London, UK). PrimeScript RT reagent Kit (TaKaRa, Otsu, Japan). Naïve CD4^+^ T Cell Isolation Kit (Miltenyi Biotec, Germany), ionomycin, and phorbol myristate acetate (PMA) were obtained from Sigma-Aldrich (St. Louis, MO, USA). Nucleofector Solution (VCA-1003) was purchased from (Lonza Amaxa, Germany). Radioimmunoprecipitation assay buffer (RIPA) and phenylmethylsulfonylfluoride (PMSF) were obtained from Beyotime (Shanghai, China).

### Mice

C57BL/6 male mice (male) were purchased from the Guangdong Medical Laboratory Animal Facility (Foshan, China). Eight-week-old animals were used. Animal care and experimental protocols were approved by the Ethics Committee of Southern Medical University.

#### Immunomagnetic bead isolation, cell culture, and Th22 differentiation

Mice were sacrificed, and the bodies were rinsed with 75 % ethanol. Subsequently, only lymph nodes without fat tissue were collected using forceps. The collected lymph nodes were put into a tissue culture dish with 4 mL of PBS with 2 % FBS and ground to obtain single-cell suspension with a syringe plunger. A stainless wire net with 200 meshes was used to filter the lymphocytes. The cells were harvested, centrifuged at 300 g for 5 min, and the supernatant was discarded. The cells were resuspended in 2 mL of red blood cell lysis buffer, and kept on ice for 5 min. The cells were then resuspended with RPMI 1640 supplemented with 10 % fetal bovine serum, 100 U/mL penicillin, 100 mg/mL streptomycin, and cell number was counted at a concentration of 1 × 10^8^ cells/mL. Finally, the buffer was discarded.

According to the manufacturer’s instructions, from separated lymphocytes, naïve CD4^+^ (CD4^+^CD62L^+^) T cells were isolated using a mouse naïve CD4^+^ T cell isolation kit. The freshly harvested naïve CD4^+^ T cells were double-stained with anti-CD4-conjugated FITC and anti-CD62L-conjugated eFluor 450 to a purity of 94 % under a flow cytometer. The cells were then cultured in RPMI 1640 complete medium and maintained at 37 °C in an incubator containing 5 % CO_2_.

The obtained purified CD4^+^ T cells were stimulated by anti-mouse CD3 Ab and anti-mouse CD28 Ab in a ratio of 1:1 (1 µg/mL), combined with factors such as IL-6 (30 ng/ml),TNF-α (50 ng/mL), IL-23 (50 ng/mL), anti-IL-4 (10 µg/mL), and anti-IFN-γ (10 µg/mL) to differentiate into Th22 cells. In a set of experiments, different concentrations of jagged-1 (0, 0.5, 1, 5 µg/mL) or the NOTCH signaling inhibitor γ-secretase inhibitor DAPT (0, 1, 5, 10 µM) were added to the culture medium for 6 days. Cells treated with anti-mouse CD3 /CD28 Ab with an equal volume of RPMI 1640 complete medium served as a control. The cells treated with a combination of factors (IL-6, TNF-α, IL-23) formed the Th22 group, and jagged-1 (1 µg/mL) or jagged-1 (1 µg/mL) plus DAPT (10 µmoL/L) treated cells formed the jagged-1 group or DAPT group for qPCR, Western blotting and flow cytometry, respectively.

#### RNA interference

The HES-1 siRNA and mismatch HES-1 siRNA were synthesized by RiboBio (Guangzhou, China). The sequences of the HES-1 siRNA and the mismatch HES-1 siRNA were 5ʹ-CGAGGUGACC CGCUUCCUGdTdT-3ʹ and 5ʹ-CGAGGUCACCCGGUUCCUGdTdT-3ʹ, respectively. Freshly naïve CD4^+^ T cells were transfected with 100 nM of HES-1-targeting siRNA or 100 nM of mismatch HES-1 siRNA (M-HES-1-siRNA) according to the manufacturer’s instructions. Subsequently, the cells were cultured in RPMI 1640 complete medium with a combination of factors that induced Th22 cells for 6 days.

#### Nuclear transfection with HES-1 overexpression vector

The obtained naive CD4^+^ T cells (1 × 10^6^ cells per sample) were resuspended in 100 µL of room-temperature Nucleofector Solution (VCA-1003) (Lonza Amaxa, Germany) according to the manufacturer’s instructions. The obtained cell suspension was combined with 2 µg of the pCVM6-HES-1 vector or 2 µg of the vector control (GenePharma, Shanghai, China). Subsequently, the naïve CD4^+^ T cells and DNA suspension were transfected into a certified cuvette without air bubbles. The Nucleofector Program X-001 was used, and the cuvette with the above mixture was inserted for transfection. The transfected or untransfected cells were treated for 6 days with a combination of factors that induced Th22 cells.

#### Flow cytometry analysis

Later, the cells were treated with different culture conditions for inducing Th22 cells (described above). Six hours before the end of the above treatment (with a combination of factors), 50 ng/mL (PMA) and 1 mg/mL of ionomycin were added to the cells. Brefeldin A (10 µg/mL) was added to block protein transportation to the Golgi complex and protein accumulation in the endoplasmic reticulum. Surface staining was performed using anti-CD4-conjugated FITC at 37 °C for 15 min. The cells were washed, resuspended, fixed, and permeabilized with fixation/permeabilization buffer according to the manufacturer’s instructions. Intracellular staining with anti-IL-17A-conjugated PE, anti-IL-22-conjugated APC, and anti-IFN-γ-conjugated PerCP-Cyanine5.5 was performed, also according to the manufacturer’s protocol. Flow cytometry was used to analyze the cells by applying a CD4^+^IFN-γ^−^ gate on a flow cytometer (FACSAria, Becton Dickinson, Franklin Lakes, NJ, USA). Experiments were performed in triplicate, and Flow Jo Version 7.6.1 was used for data analysis.

For some experiments, naïve CD4^+^ T cells cultured in Th22 cell culture conditions and treated with 1 µg/mL jagged-1 and 10 µM DAPT were used. For some experiments, naïve CD4^+^ T cells were transfected with M-HES-1-siRNA, HES-1-siRNA, vector control, and pCMV6-AC-HES-1, with a combination of factors that induced Th22 cells as in the previous description. Flow cytometry was used to analyze the percentage of Th22 cells.

#### Quantitative real‐time PCR

The total RNA was extracted from different treatment cells using Trizol Reagent, according to the manufacturer’s instructions. Reverse transcription (RT) was performed in a 20 µL reaction system using a TaKaRa PrimeScript reagent kit with the gDNA eraser (TaKaRa, Otsu, Japan) to synthesize cDNA, according to the manufacturer’s recommendation. The cDNA was then amplified with a PrimeScript RT reagent kit according to the manufacturer’s instructions. Primers used in this study are listed as follows: *Nicd* sense 5ʹ-TGAATGGCGGGAAGTGTGAA-3ʹ, *Nicd* antisense 5’-ATAGTCTGCCACGCCTCTG-3, *Il-22* sense 5ʹ-ATGAGTTTTTCCCTTATGGGGAC-3ʹ, *Il-22* antisense 5ʹ-GCTGGAAGTTGGACACCTCAA-3ʹ, *Ahr* sense 5ʹ- CAAATCCTTCCAAGCGG.

CATA-3ʹ, *Ahr* antisense 5ʹ-CGCTGAGCCTAAGAACTGAAAG − 3ʹ, *Hes-1* sense 5ʹ-CAGCCAGTGTCAACACGACACCGGACAAAC-3ʹ, *Hes-1* antisense 5ʹ-TGCCCTTCGCCT.

CTTCTCCATGATA-3ʹ, *β-actin* sense 5ʹ-AACAGTCCGCCTAGA AGCAC-3ʹ, *β-actin* antisense 5ʹ-CGTTGACATCCGTAAAGACC-3ʹ. Fluorescence was detected using a CFX96 Touch instrument (Bio-Rad, Hercules, CA). Each sample was run in triplicate and was compared with *β-actin* as the reference gene. Results were analyzed by the 2^−ΔΔCT^ method for the relative quantification of mRNA expression.

### Western blotting analysis

Cells from the treatment and control groups were harvested, and washed once with cold PBS for total protein extraction. The cells were lysed with RIPA containing 1 mM PMSF for 20 min on ice. Then, the lysates were centrifuged (12,000 × g 30 min at 4 °C). The supernatants were transferred to new tubes. Bicinchoninic acid (BCA) assay was used to determine protein concentration. Then, the sample was denatured by boiling it at 95℃ for 5 min with a loading buffer. The protein analysis was carried out on 12 % sodium dodecyl sulfate polyacrylamide gel electrophoresis (SDS-PAGE) gels and transferred electrophoretically to polyvinylidene difluoride (PVDF) membranes. After blocking with 5 % bovine serum albumin (BSA) dispensed with Tris-buffer saline containing 0.1 % Tween-20 (TBST) for 1 h at room temperature, the PVDF membranes were incubated overnight at 4 °C with the indicated primary antibodies: anti-STAT3 (1:1000, #sc-8019, Santa Cruz), anti-p-STAT3 (1:1000, #sc-8059, Santa Cruz), anti-NICD (1:1000, #sc-32,745, Santa Cruz), anti-HES-1 (1:2000, #ab108937, Abcam), anti-AHR (1:2000, #ab85666, Abcam), anti-IL-22 (1:2000, # ab134035, Abcam) and anti-Actin (1:1000,#AA128, Beyotime). The membranes were washed three times with TBST and then incubated with the appropriate horseradish peroxidase (HRP)-conjugated secondary antibody for 1 hour at room temperature. An enhanced chemiluminescence detection kit (#35,050, Thermo Fisher) was used to visualize specific bands on the membranes according to the manufacturer’s instructions. ChemiDoc Touch (Bio-Rad, Hercules, CA) was used to quantify the band density. Quantity One analysis software (Bio-Rad, Hercules, CA) was used to analyze the protein band.

#### Statistical analysis

Statistical analysis of data was performed using GraphPad Prism 6.0 (GraphPad Software Inc., San Diego, CA, United States). Student’s t test was used to compare two groups. Non-parametric one-way analysis of variance (ANOVA) followed by Tukey’s *post hoc* test was used to analyze the statistical significance among multiple groups. Results are expressed as the mean ± SD, with *p* < 0.05 being considered as statistically significant. All experiments were performed at least three times independently, with one representative experiment shown here.

## Results

### IL-6, TNF-α, and IL-23 treatment promoted differentiation of naïve CD4^+^ T cells into Th22 cells

**Fig. 1:**
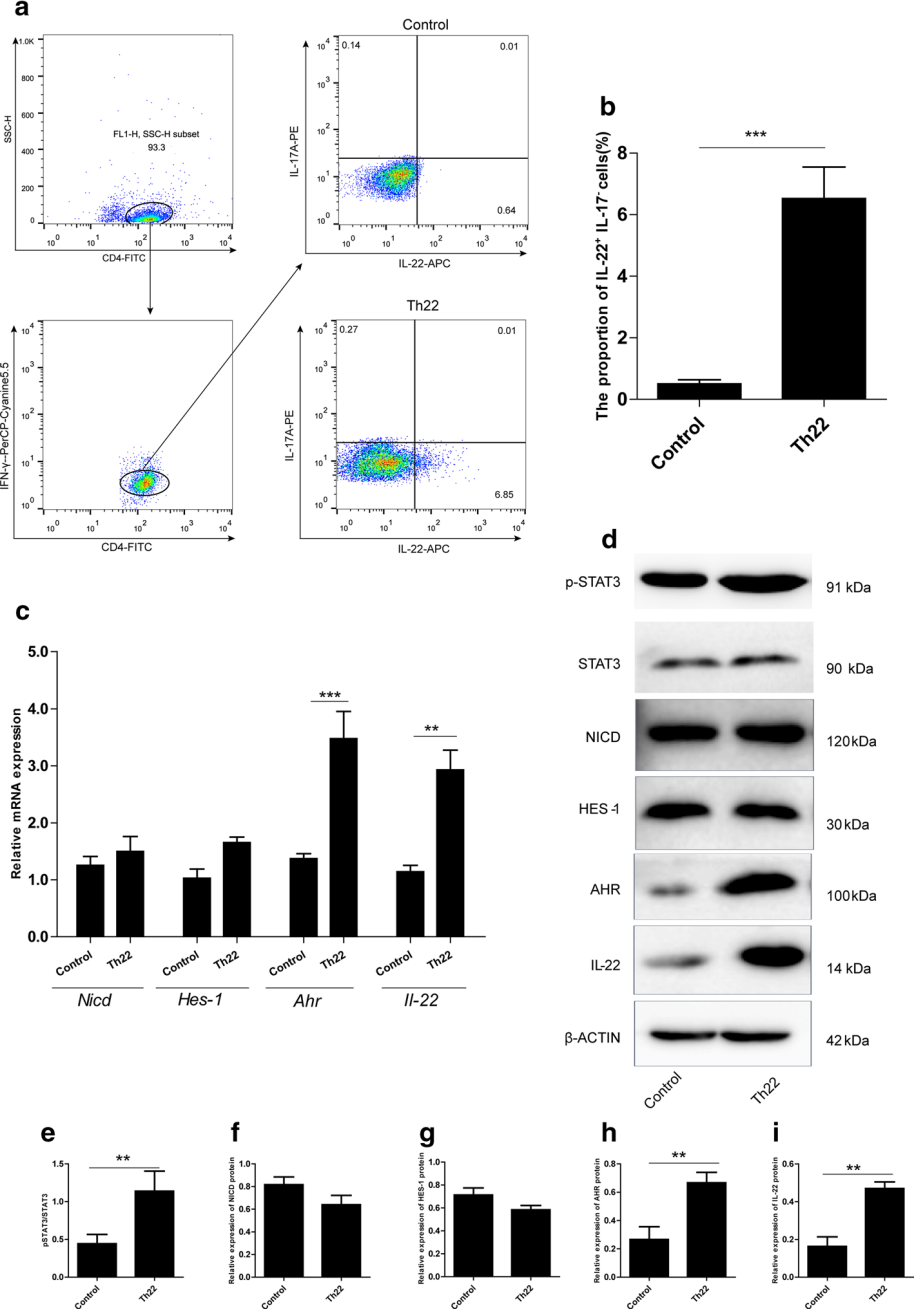
Naïve CD4^+^ T cells could be differentiated into Th22 cells with multiple cytokines in vitro. Naïve CD4^+^ T cells were collected from mice and cultured for 6 days in conditions designed to induce Th22 differentiation (anti-CD3/CD28 Abs, anti-IFN-γ Ab, anti-IL-4 Ab, IL-6, TNF-α, and IL-23). **a** Representative plots of naïve CD4^+^T cells stimulated for 6 days under optimal Th22 conditions. CD4^+^ T cells and Th (CD4^+^ IFN-γ^−^) were gated by flow cytometry to analyze the Th22 cells. **b** Percentage quantitation of Th22 cells. **c** The alterations of *Nicd*, *Hes-1*, *Ahr*, and *Il-22* mRNAs were evaluated by RT-PCR. **d** Western blotting of the expression of p-STAT3, STAT3, NICD, HES-1, AHR, and IL-22 in total protein lysates from different treatment cells. e–i Representative densitometric quantification of p-STAT3, STAT3, NICD, HES-1, AHR, and IL-22 expression in T cells, β-ACTIN was used as an endogenous control for protein expression. The results show a typical experiment; each bar represents the mean ± S.E.M. of at least three independent experiments. **p < 0.01, ***p < 0.001, compared with control group

Immunomagnetic separation was used to isolate and purify naïve CD4^+^ T cells. First, polarized Th22 (CD4^+^IL22^+^IFNγ^−^IL17A^−^) cells were established by culturing purified naïve CD4^+^ T cells which were treated with anti-mouse CD3/CD28, anti-IL-4, anti-IFN-γ antibodies, simultaneously with a combination of factors (IL-6, TNF-α, and IL-23) *in vitro*. Later, extracellular and intracellular staining of the cells was done to investigate the frequencies of Th22 cells. The obtained results showed that after the presence of a combination of factors on the naïve CD4^+^ T cells, they were obviously converted into Th22 cells compared with the control (p < 0.001 ) (Fig. 1a, b). As assessed by RT PCR, mRNA expression of *Ahr* and *Il-22* significantly increased after the treatment with a combination of factors compared with the control (*p*<0.001, *p*<0.01) (Fig. 1c). However, treatment with a combination of factors did not affect the mRNA expression of *Nicd* and *Hes-1* when compared with the control (*p* > 0.05) (Fig. 1c). Furthermore, the western blotting analysis was used to analyze the expression of p-STAT3, STAT3, NICD, HES-1, AHR, and IL-22. The protein expression was consistent with the expression seen in the qPCR analysis. After treatment with the combination of factors, both AHR and IL-22 expression levels were significantly increased (*p*>0.001) (Fig. 1d, h − i), and the activity of STAT3 was enhanced (Fig. 1d, e). However, treatment with a combination of factors did not affect the expression levels of NICD and HES-1, when compared with control treatment (*p *> 0.05) (Fig. 1d, f − g). Treatment with a combination of factors elevated p-STAT3, AHR and IL-22 compared with the control. These results show that IL-6, TNF-α, and IL-23 might strongly activate the STAT3 pathway, and promote the differentiation of naïve CD4^+^ T cells into Th22.

### NOTCH signaling governed IL-22 expression

**Fig. 2:**
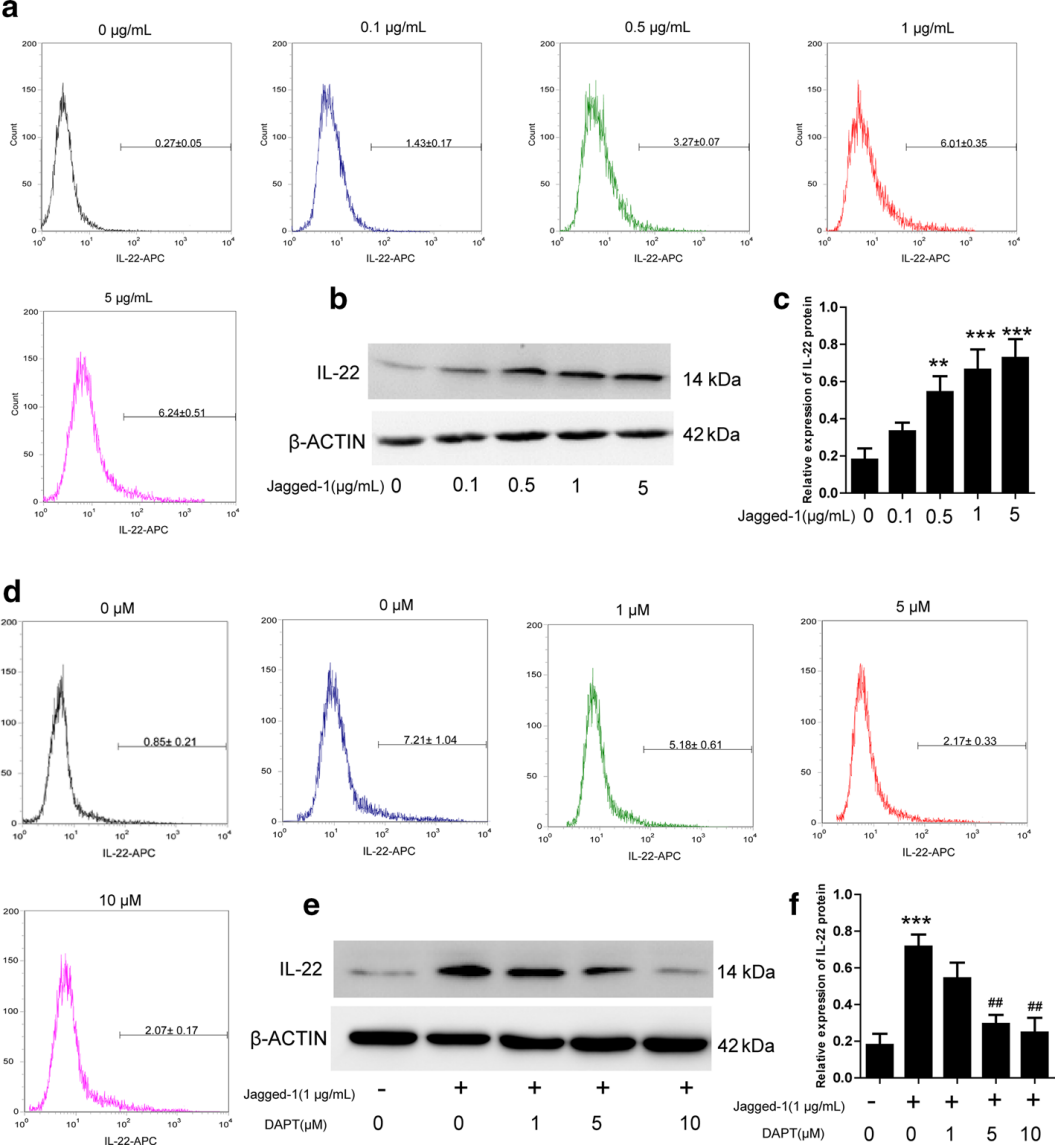
Effect of different concentrations of jagged-1 and DAPT on mediated expression of IL-22. After the naïve CD4^+^ T cells were pretreated with a combination of factors, different concentrations of jagged-1 (0, 0.1, 0.5, 1, 5 µg/mL) were added to culture medium. **a** Flow cytometric analysis of the positive IL-22 cell proportion in different doses of jagged-1 treatment. **b**, **c** IL-22 proteins were determined by Western blotting. **d** Naïve CD4^+^ T cells were treated with the combination of factors, and exposure with or without jagged-1 (1 µg/mL), and different doses of DAPT added (0, 1, 5, 10, 20 µM). Then, the percentages of positive IL-22 cells were assayed by flow cytometry. **e**, **f** Following the treatment, expression of IL-22 protein was determined by Western blotting. Data are presented as the mean ± SD of three independent experiments. **p < 0.01, ***p < 0.001 vs. untreated control, ^##^*p* < 0.01 vs. jagged-1 (1 µg/mL) without DAPT group

To investigate the role of NOTCH signaling in CD4^+^ T cells expression of IL-22 was assessed. The purified naïve CD4^+^ T cells were treated with various cytokines as mentioned above, and then exposed to jagged-1 at various concentrations (0, 0.5, 1, 5 µg/mL) for 6 days. Subsequently, flow cytometry and western blotting were performed to analyze the cells and protein of IL-22. The proportion of IL-22^+^ cells gradually increased with increasing jagged-1 concentration (Fig. 2a). A concentration-dependent increase in the level of IL-22 was also detected in naïve CD4^+^ T cells exposed to jagged-1 (Fig. 2b, c). Since the main objective of this study was to identify the change in IL-22 expression rather than toxicity, an exposure dose of up to 1 µg/mL was used as the optimum concentration for inducing IL-22.

To further confirm the role of NOTCH signaling in the expression of IL-22, we used DAPT (0, 1, 5, 10 µM), a γ-secretase inhibitor, which suppresses NOTCH signaling, to analyze the alteration of IL-22. The results demonstrated that DAPT could reduce the proportion of IL-22^+^ cells, and reduce the level of IL-22 in a concentration-dependent manner (Fig. 2d−f). A 10 µM concentration of DAPT was sufficient for inhibiting the expression of IL-22. Thus, the activation of NOTCH signaling with jagged-1 increased the level of IL-22 in a dose-dependent manner, and blockade with DAPT resulted in it declining, also in a dose-dependent manner.

### Blocked NOTCH signaling pathway inhibited Th22 cell polarization

**Fig. 3:**
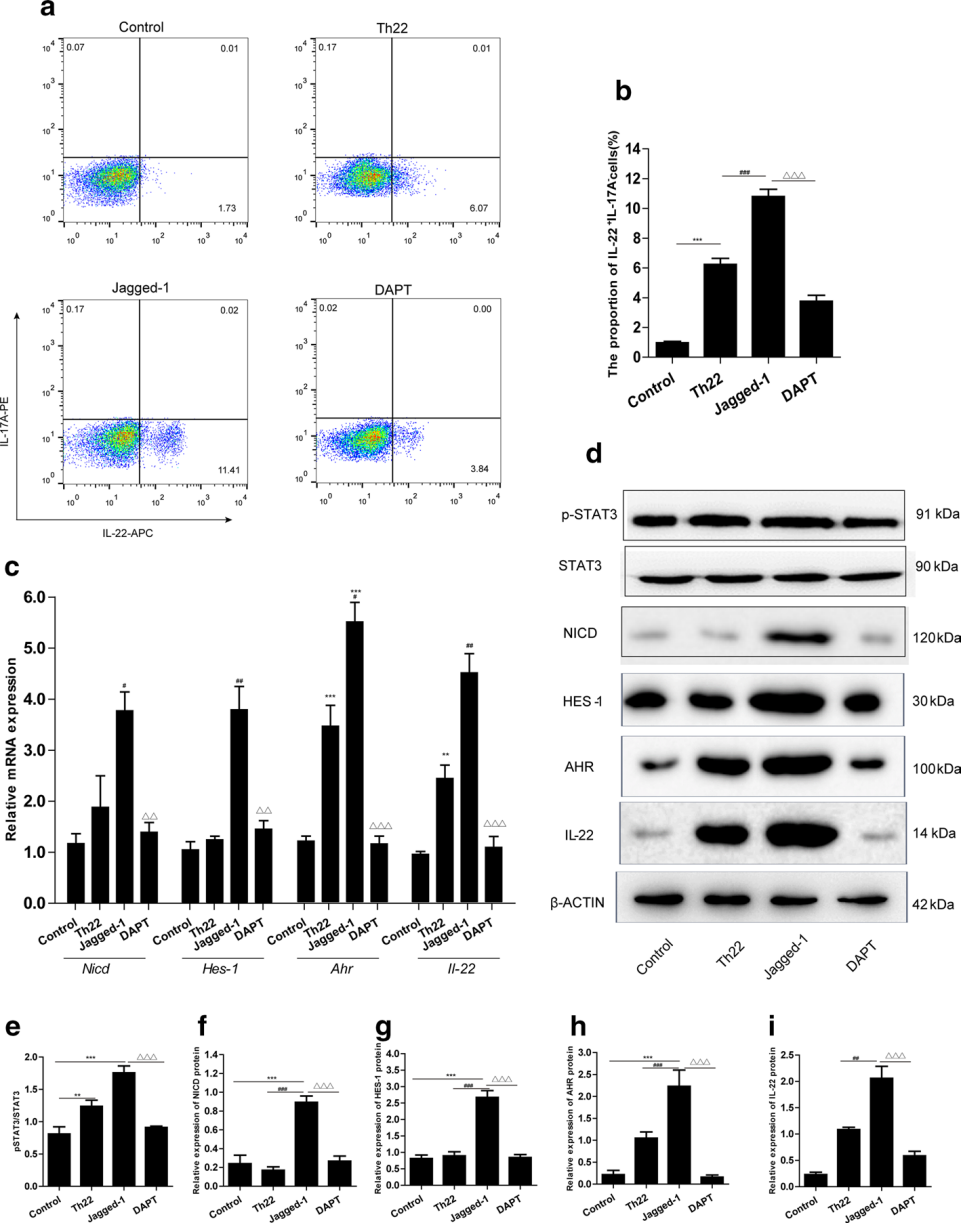
NOTCH signaling pathway was involved in differentiation of CD4^+^ T cells towards Th22 cells. Naïve CD4^+^ T cells were cultured in conditions designed to induce Th22 differentiation, either 1 µg/mL jagged-1 treatment alone or jagged-1 combined with DAPT (10 µM). **a** The percentage of Th22 (CD4^+^IL-22^+^IFNγ^−^IL-17ˉ) cells was analyzed by flow cytometry in naïve CD4^+^ T cells treated with PBS, jagged-1 or jagged-1 plus DAPT. **b** Percentage quantitation of Th22 cells. **c** Gene expression of *Nicd*, *Hes-1*, *Ahr*, and *Ii-22* was analyzed by RT-PCR. **d** Representative western blot showing protein levels of p-STAT3, STAT3, NICD, HES-1, AHR, and IL-22 extracted from different groups. **e**−**i** Densitometric analysis of p-STAT3, STAT3 NICD, HES-1, AHR, and IL-22 levels was performed with Quantity One software and data were represented as the means ± S.E.M. of three different experiments. **p < 0.01, ***p < 0.001, compared with control group; ^#^*p* < 0.5, ^##^*p* < 0.01, ^###^*p* < 0.001, for jagged-1 versus Th22 groups; ^△△^*p* < 0.01, ^△△△^*p* < 0.001, for DAPT versus jagged-1 groups

To address the involvement of possible molecular mechanisms with NOTCH signaling in the polarization of Th22 cells, jagged-1 (1 µg/mL) and DAPT (10 µM) were used. Flow cytometry was performed to analyze the effect of phenotypes of CD4^+^ T cells treated with jagged-1 alone or jagged-1 combined with DAPT on the frequency of Th22 cells. Treatment with a combination of factors significantly increased the percentage of Th22 cells, and jagged-1 further increased it (Fig. 3a, b), whereas treatment with jagged-1 plus DAPT markedly decreased the proportion of Th22 compared to the jagged-1 group (Fig. 3a, b). Simultaneously, this treatment also inhibited the mRNA expression of *Nicd*, *Hes-1*, *Ahr*, and *Il-22* (Fig. 3c), and significantly reduced the protein levels of p-STAT3, NICD, HES-1, AHR, and IL-22 (Fig. 3d − f) compared with the jagged-1 group (*p*<0.001, *p*<0.05). Thus, these findings provided further evidence that NOTCH signaling plays an important role, and inhibiting it would contribute to a reduction in the differentiation of naïve CD4^+^ T cells towards Th22 cells.

### HES-1 plays an essential role in mediating naïve CD4^+^ T cell differentiation into Th22

**Fig. 4:**
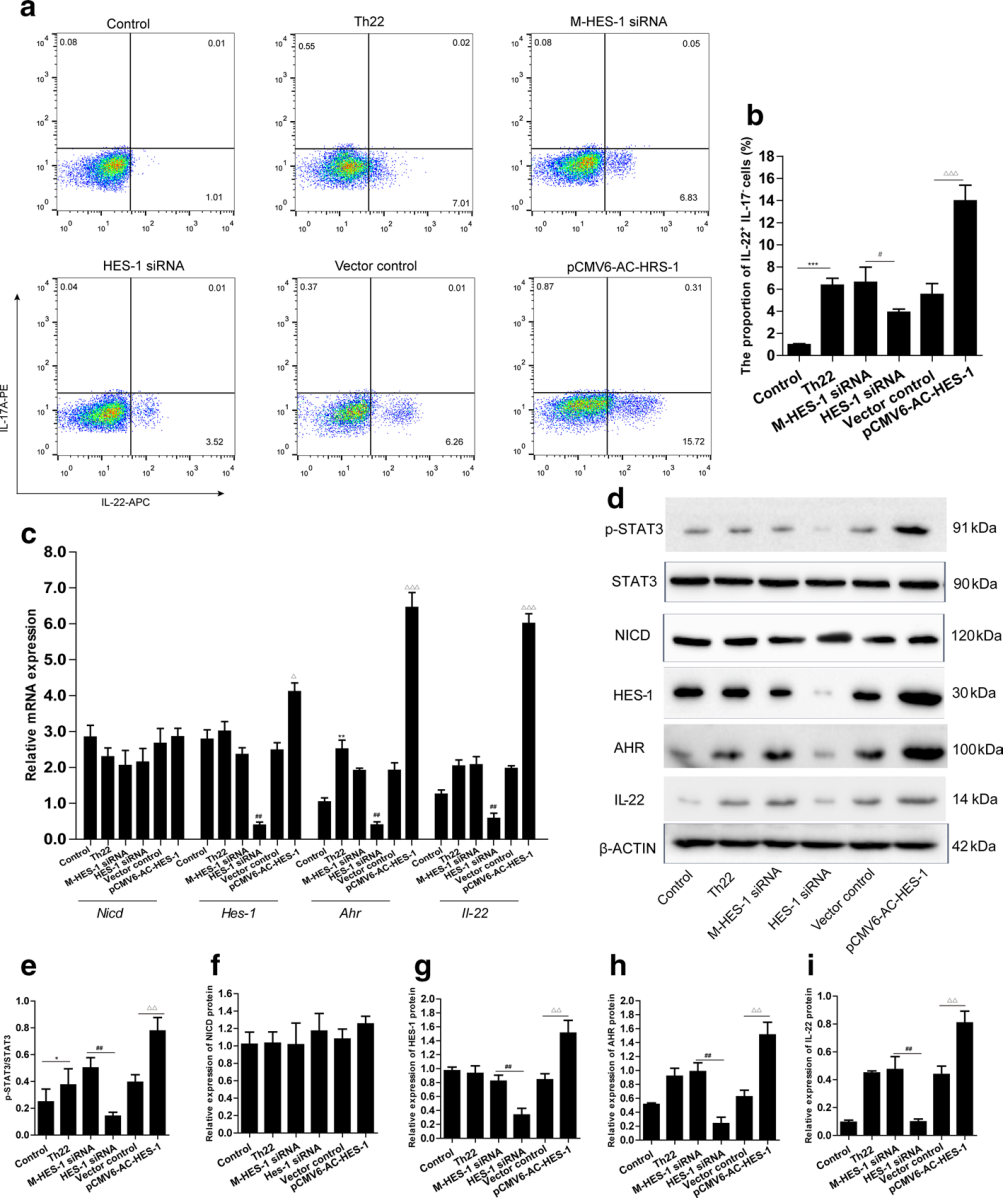
HES-1 was indispensable in naïve CD4^+^ T cell differentiation into Th22 cells. Naïve CD4^+^ T cells were transfected with HES-1-siRNA, HES-1 plasmid, or their controls. Subsequently, the transfection cells were cultured in conditions designed to induce Th22 differentiation. **a** The intracellular IL-17A and IL-22 in transfection CD4^+^ T cells were assayed by flow cytometry 6 days after the combination of factors induced Th22 cells. **b** Quantitative analysis of the percentage of IL-22^+^ IL-17A^−^ cells was performed. **c** RT-PCR was performed to evaluate the expression of *Nicd*, *Hes-1*, *Ahr*, and *Il-22.* **d** Western blot for p-STAT3, STAT3, NICD, Hes-1, AHR, and IL-22 was performed to assess the alteration. **e**−**i** The relatively quantified protein bands from western blot were executed with the Quantity One software and data were represented as the means ± S.E.M. of three different experiments. *p < 0.05, ***p < 0.001, compared with the control group; ^#^*p* < 0.5, ^##^*p* < 0.01, for HES-1 siRNA versus M-HES-1siRNA groups; ^△^*p* < 0.05, ^△△^*p* < 0.01, ^△△△^*p* < 0.001, for pCMV6-AC-HES-1 versus vector control groups

HES-1 is a downstream target gene of the NOTCH signaling pathway [28]. We further explored whether alteration of HES-1 expression affects the proportions of naïve CD4^+^ T cells to Th22 cells. Naïve CD4^+^ T cells were transfected with HES-1 plasmid or HES-1-siRNA and then exposed to the combination of factors for inducing Th22 cells as described in the "Materials and Methods" section. The results show that both HES-1 siRNA and HES-1 plasmid were able to remarkably alter the expression of HES-1 in naïve CD4^+^ T cells (*p*<0.001, *p*<0.05) (Additional file 1: Fig. 1a, b). The percentage of Th22 cells was analyzed by flow cytometry. We observed that knock down of the *Hes-1* gene with HES-1 siRNA resulted in the reduction of Th22 frequency, whereas overexpression of HES-1 significantly promoted the elevation of Th22 cells (*p*<0.001, *p*<0.05) (Fig. 4a, b). Compared with the M-HES-1 siRNA group the HES-1 siRNA treatment not only significantly suppressed the level of *Hes-1*, but also reduced the expression of *Ahr* and *Il-22* (*p*<0.01) (Fig. 4c). In contrast, as assessed by qRT-PCR, the overexpression of HES-1 was able to boost the level of *Hes-1*, *Ahr*, and *Il-22* levels compared to the vector control (*p*<0.01, *p*<0.001) (Fig. 4c). We also found that no matter how high or low, the expression of HES-1 did not affect the level of NICD (p > 0.05) (Fig. 4c−d, f), but the level of p-STAT3 could be changed (Fig. 4d, e). The total protein levels of HES-1, AHR, and IL-22 were consistent with the changes observed in their mRNA expression (*p*<0.01) (Fig. 4c, g−i). These results demonstrated that the NOTCH signaling pathway downstream target gene *Hes-1* plays a role in the differentiation of naïve CD4^+^ T cells into Th22 cells.

## Discussion

Various studies have reported that Th22 cells are a new subset of Th cells that are phenotypically different from Th17 cells [11, 29]. Th22 produces only high levels of IL-22 and neither IL-17A nor interferon-γ [9, 29]. A previous study showed that a combination of four factors and an inhibitor [IL-6, IL-23, IL-1β, FICZ (aryl hydrocarbon receptor ligands), and TGF-βR inhibitor] could significantly differentiate naïve CD4^+^ T cells into Th22 cells [29]. This treatment increased the expression of IL-22 significantly and suppressed the production of IL-17A. They also found that IL-6, IL-23, and IL-β increased the production of both IL-22 and IL-17. However, a study by Thomas Duhen [9] showed that treatment with IL-6 alone or combined stimulation with IL-6 and TNF-α initiated the differentiation of Th22 cells and elevated the production of IL-22. When the combined stimulation with IL-1β added further enhanced IL-22, IL-17 was also strongly induced at the same time. Furthermore, the addition of high doses of TGF-β markedly suppressed the differentiation of Th22 cells. The study of autoimmune diseases such as rheumatoid arthritis has shown that a combination of TNF-α, IL-1β, and IL-6 effectively promotes the differentiation of Th22 cells [30]. Although many cytokines have been suggested to induce naïve CD4^+^ T cells to express IL-22 [9, 29, 31], this has not been achieved. Based on previous studies, we employed IL-6, IL-23, and TNF-α combined stimulation of naïve CD4^+^ T cells to induce their differentiation into Th22 cells. Flow cytometry analysis of the naïve CD4^+^ T cells exposed to TNF-α, IL-6, and IL-23 showed that this treatment could obviously increase the proportion of Th22 cells, up-regulate the expression of AHR and IL-22, and cause phosphorylation of STAT3. Therefore, the role of TNF-α, IL-6, and IL-23 in inducing Th22 cells is further verified.

The NOTCH signaling pathway plays a significant role in the modulation and differentiation of T cells [32]. Previous studies have suggested that NOTCH signaling mediates the production of IL-22 from CD4^+^ T cells [14, 26, 33]. Muhammad Shamsul Alama et al. found that the level of IL-22 increased due to overexpression of NICD or NOTCH ligand in CD4^+^ T cells, and the expression of IL-22 remained unaltered by γ-secretase inhibitor treatment or RBP-J-deficient, *in vitro* and *in vivo*[14]. This study showed that treatment of naïve CD4^+^ T cells with a combination of factors promoted the differentiation of Th22 cells, and the addition of the NOTCH signaling ligand jagged-1 further enhanced the proportion of Th22 cells, as well as elevating the mRNA and protein level of AHR and IL-22. Our findings are in accordance with those of Muhammad Shamsul Alama, who showed that the NOTCH signaling pathway promotes the differentiation of Th22 cells.

In contrast, pharmacological inhibitors were used to block this pathway, with DAPT downregulating the proportion of Th22 cells, along with NICD and HES-1 mRNA and protein, transcription factor AHR and IL-22. Similarly, studies on lung adenocarcinoma and hepatitis C virus-infected patients also support the observation that activated NOTCH signaling increases the percentage of Th22 cells [26, 33]. Furthermore, it was proposed that the NOTCH-AHR-IL-22 axis was involved in this process. Consistent with this finding, our study indicated that the expression of both AHR and IL-22 was up-regulated in the Th22 and jagged-1 groups after activation of NOTCH signaling. Interestingly, upregulation of the NOTCH signaling target protein HES-1 was also observed, and expression of NICD, HES-1, AHR, and IL-22 was reduced by DAPT. This study focused on the NOTCH signaling downstream target protein HES-1 to demonstrate its role in mediating naïve CD4^+^ T cells’ differentiation into Th22 cells.

**Fig. 5:**
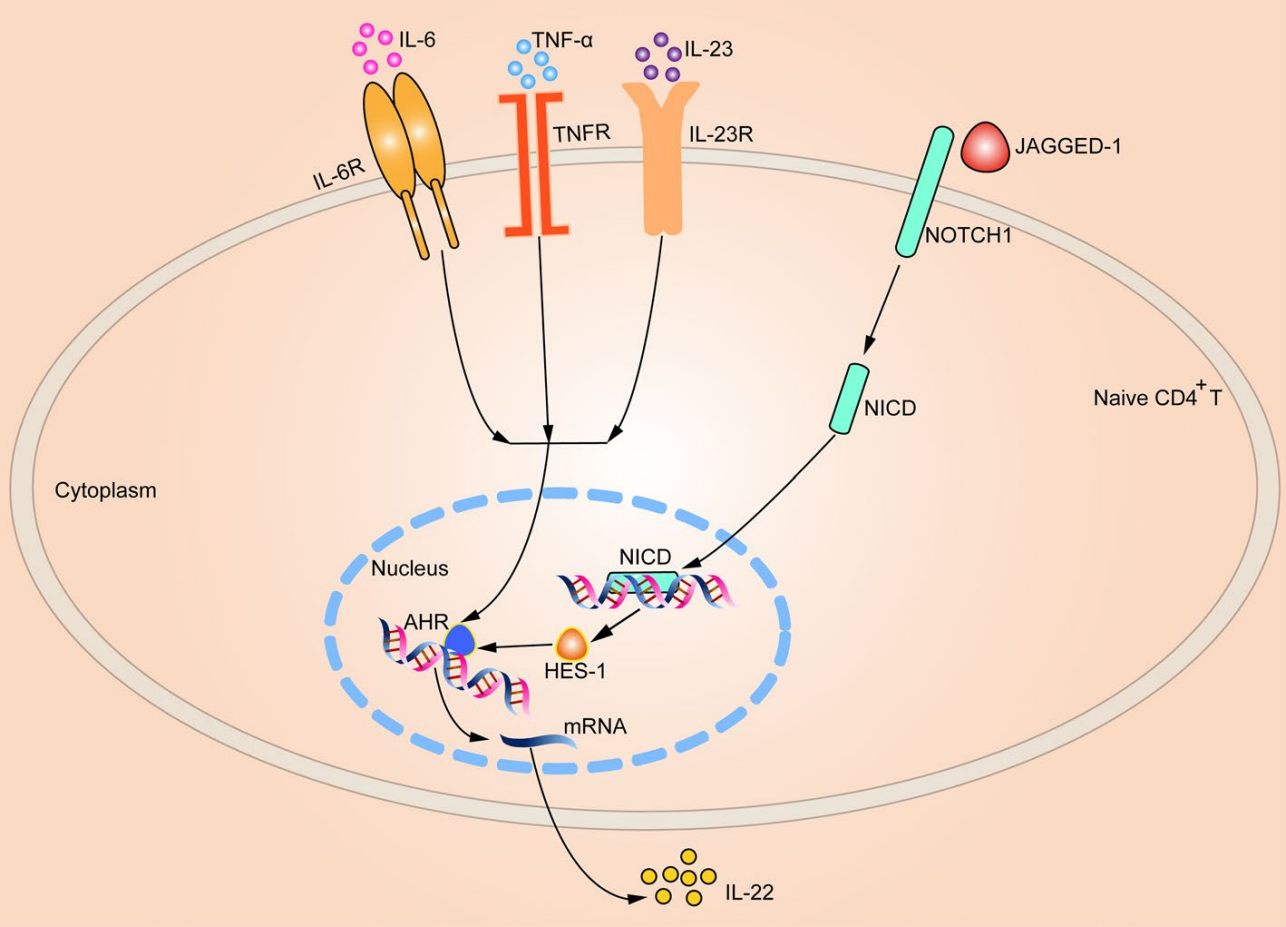
Schematic diagram illustrating the induction mechanism of Th22 cell differentiation, with involvement of NOTCH signaling

According to a previous study, retroviral overexpression of the NOTCH signaling downstream target gene *Hes-1* does not influence T cell differentiation in human CD34^+^ hematopoietic stem cells [34]. However, a later study reported that inhibition of NOTCH1 signaling with DAPT could decrease the population of splenic Th17 cells, mRNA expression of Th17 cell-specific transcription factor RORγt, Hes-1, as well as IL-17A mRNA and IL-17A serum concentration in a mouse model of psoriasis-like skin inflammation [35]. In addition, another study [32] also supported the view that activating NOTCH signaling would promote the differentiation of Th17 cells in experimental autoimmune encephalomyelitis. Hence, there might be no consensus on the effect of NOTCH signaling in T cells’ development and differentiation. In this study we focused on Th22 cells, which showed that overexpression of HES-1 improved the proportion of Th22 cells as well as the mRNA and protein expression of AHR and IL-22, and promoted STAT3 phosphorylation. On the other hand, *Hes-1* knockdown with siRNA reduced the percentage of Th22 and inhibited both AHR and IL-22, and decreased STAT3 phosphorylation. Furthermore, neither overexpression nor knockdown of HES-1 could alter the mRNA and protein expression of NICD. Thus, the results of this study suggest the possibility of the role of NOTCH signaling and the downstream gene *Hes-1* in regulating Th22 cells. A schematic diagram showing the action of IL-6, IL-23, and TNF-α in inducing naïve CD4^+^ T cells into Th22 cells with NOTCH signaling is shown in Fig. 5. However, a limitation of this study is that we have not precisely explored the role of the STAT3 signaling pathway in this process.

## Conclusions

This study indicates that the NOTCH signaling pathway plays an essential role in the development and differentiation of T cells, especially Th22 cells. Activated NOTCH signaling or overexpression of the NOTCH downstream target HES-1 induces naïve CD4^+^ T cells toward differentiation of Th22. These results expand our knowledge about the NOTCH signaling in T cells and may provide an additional therapeutic intervention approach to autoimmune disease.

## Supplementary Information

**Additional file 1.** WB results showed SHARPIN expression after transfection with SHARPIN siRNA and plasmid.

## Data Availability

Datasets used and/or analyzed in this study can be obtained from the corresponding author on reasonable request.

## Abbreviations

TNF-α: Tumor necrosis factor-α
IL-22: Interleukin-22
DAPT: (2S)-N-[(3,5-difluorophenyl) acetyl]-L-alanyl-2-phenyl] glycine1, 1-dimethylethyl ester
NICD: NOTCH intracellular domain
TGF-β: Transforming growth factor-β
HES-1: Hairy and enhancer of split 1
RORγt: Retinoid-related orphan receptor-γt
AHR: Aryl hydrocarbon receptor
FITC: Fluorescein isothiocyanate
PMA: Phorbol myristate acetate
