# Supplementary figures and images for: The NOTCH-HES-1 axis is involved in promoting Th22 cell differentiation
Source: Cell Mol Biol Lett. 2021 Feb 23;26:7. doi: 10.1186/s11658-021-00249-w (PMC7901075; doi:10.1186/s11658-021-00249-w)

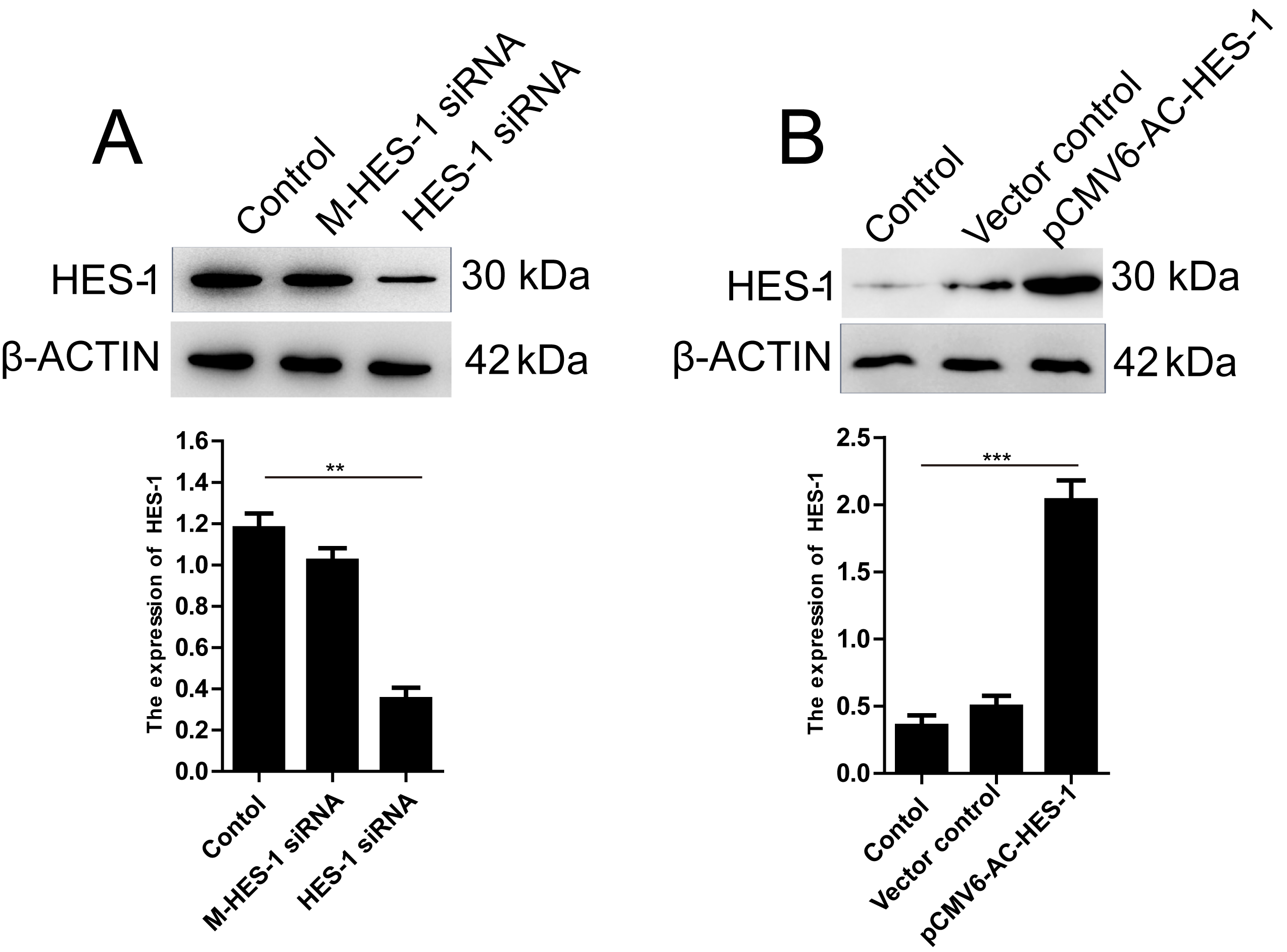

Supplement: Supplementary file 1 — Additional file 1. WB results showed SHARPIN expression after transfection with SHARPIN siRNA and plasmid. [file 11658_2021_249_MOESM1_ESM.tif]
